# An analysis of microbiota-targeted therapies in patients with avian influenza virus subtype H7N9 infection

**DOI:** 10.1186/1471-2334-14-359

**Published:** 2014-07-02

**Authors:** Haifeng Lu, Chunxia Zhang, Guirong Qian, Xinjun Hu, Hua Zhang, Chunlei Chen, Weifeng Liang, Hainv Gao, Yunmei Yang, Lanjuan Li

**Affiliations:** 1 State Key Laboratory Diagnosis and Treatment for Infectious Disease, Collaborative Innovation Center for Diagnosis and Treatment of Infectious Diseases, the First Affiliated College of Medicine, Zhejiang University, 79 Qingchun Road, Hangzhou 310003, China; 2 Collaborative Innovation Center for Diagnosis and Treatment of Infectious Diseases, Hangzhou, China; 3 Department of Geriatrics, the First Affiliated Hospital, College of Medicine, Zhejiang University, 79 Qingchun Road, Hangzhou 310003, P.R. China

**Keywords:** H7N9, Intestinal microbiota, Probiotics, Microbiota restoration treatment, Quantitative PCR

## Abstract

**Background:**

Selective prophylactic decontamination of the digestive tract is a strategy for the prevention of secondary nosocomial infection in patients with avian influenza virus subtype H7N9 infection. Our aim was to summarize the effectiveness of these therapies in re-establishing a stable and diverse microbial community, and reducing secondary infections.

**Methods:**

Comprehensive therapies were dependent on the individual clinical situation of subjects, and were divided into antiviral treatment, microbiota-targeted therapies, including pro- or pre-biotics and antibiotic usage, and immunotherapy. Quantitative polymerase chain reaction and denaturing gradient gel electrophoresis (DGGE) were used for real-time monitoring of the predominant intestinal microbiome during treatment. Clinical information about secondary infection was confirmed by analyzing pathogens isolated from clinical specimens.

**Results:**

Different antibiotics had similar effects on the gut microbiome, with a marked decrease and slow recovery of the *Bifidobacterium* population. Interestingly, most fecal microbial DGGE profiles showed the relative stability of communities under the continual suppression of the same antibiotics, and significant changes when new antibiotics were introduced. Moreover, we found no marked increase in C-reactive protein, and no cases of bacteremia or pneumonia, caused by probiotic use in the patients, which confirmed that the probiotics used in this study were safe for use in patients with H7N9 infection. Approximately 72% of those who subsequently suffered exogenous respiratory infection by *Candida* species or multidrug-resistant *Acinetobacter baumannii* and *Klebsiella pneumoniae* were older than 60 years. The combination of probiotics and prebiotics with antibiotics seemed to fail in these patients.

**Conclusions:**

Elderly patients infected with the influenza A (H7N9) virus are considered a high-risk group for developing secondary bacterial infection. Microbiota restoration treatment reduced the incidence of enterogenous secondary infection, but not exogenous respiratory infection. The prophylactic effects of microbiota restoration strategies for secondary infection were unsatisfactory in elderly and critically ill patients.

## Background

Forty cases of confirmed avian influenza virus subtype H7N9 infection were treated at the First Affiliated Hospital of Zhejiang University (China) during March–April 2013. Most of the patients were critically ill and required admission to an intensive care unit [1], wheretreatment can be divided into antiviral treatment, microbiota-targeted therapies, and immunotherapy. Secondary invasive bacterial infections associated with H7N9 infection can cause severe and fatal complications, and appropriate empirical antibiotic treatment for hospital-acquired bacterial infections is required. The human gut harbors a complex microbiome that plays a fundamental role in host health through its involvement in nutritional, immunological, and physiological functions [2]. Reciprocal interactions between intestinal microbiota and the human mucosal immune response influence the development of disease through activation of innate and adaptive immune responses [3]. Antibiotics have a profound disruptive effect on the intestinal microbiome [4], which is frequently accompanied by colonization of pathogenic microbes and dysbiosis of the host immune system, contributing to the development of potentially serious diseases [5,6]. Therefore, microbiota restoration strategies, which allow beneficial bacteria to thrive, eliminate colonization by opportunistic pathogens, and enhance resistance to intestinal colonization, are required for treatment of H7N9 infection. Intestinal colonization resistance is defined as the resistance to colonization by ingested bacteria or inhibition of overgrowth of resident bacteria normally present at low levels within the intestinal tract [7]. Probiotics and prebiotics have a beneficial effect on regeneration and restoration of microbiome homeostasis [8]. Additionally, more recent data show that probiotics can modulate host immunoregulation, alleviate intestinal inflammation, normalize gut mucosal dysfunction, and downregulate hypersensitivity reactions through control of proinflammatory and anti-inflammatory cytokines [9]. Theoretically, targeted microbiota-regulating treatment could prevent or alleviate the complications of H7N9 infection.

CBM588 (*Clostridium butyricum* 588 strain MIYA-BM tablets; Miyarisan Pharmaceutical, Tokyo, Japan) prevents antibiotic-associated diarrhea [10] and has anti-inflammatory effects in the colon as a result of butyric acid production [11]. We retrospectively assessed the effects of microbiota restoration strategies on restoring intestinal homeostasis under antibiotic exposure, and on reducing the risk of secondary infection, by real-time monitoring of the shift in predominant intestinal bacteria using quantitative polymerase chain reaction (qPCR) and PCR-based denaturing gradient gel electrophoresis (PCR-DGGE).

## Methods

### Patients and stool specimen sampling

The study was conducted between 1 April and 10 May 2013 in the First Affiliated Hospital, College of Medicine of Zhejiang University, China. Approval for the collection of patient stool samples was obtained from the ethical board of the hospital. All patients voluntarily joined the study and gave their informed consent. We prospectively studied all consecutive, nonselected inpatients who had episodes of influenza virus H7N9 infection. Patients were diagnosed according to the criteria of the Centers for Disease Control, USA, in 2013 [12]. Stool samples were collected daily, and intestinal microecology was investigated in all specimens. Patients were excluded if they provided fewer than four stool specimens during the follow-up period. The follow-up for each patient ended once their major method of feeding changed, for example when patients that had been fed using a nasogastric tube graduated to autonomous feeding. Fecal samples were collected in sterile bags, refrigerated, and immediately taken to the laboratory where they were aliquoted into 200-mg samples, frozen in liquid nitrogen, and stored at -75°C. Twenty-five patients completed sampling, and a total of 205 specimens were subject to qPCR and DGGE analysis. Eleven of these patients had secondary infection, of which, eight patients were over 60 years of age. Patient characteristics were collected from medical records and are in shown Table 1. Patients were classified into five groups according to microbiota-targeted treatment: Group A, two patients without any microbiota-targeted treatment during follow-up; Group B, one patient with one antibiotic only; Group C, six patients with probiotic only; Group D, eight patients with one antibiotic and one probiotic; and Group E, eight patients with two or more antibiotics and probiotics or prebiotics. Groups A–C had mild disease and could feed independently, whereas Groups D and E were critically ill and required nasogastric feeding. Twenty-five healthy age- and sex-matched controls (Table 1) were enrolled and provided only one stool sample. The participants did not have any organic diseases, none withdrew or were omitted from the study, and none received antibiotics, probiotics, or prebiotics during the sampling period.

**Table 1:** Characteristics of all subjects andusage of microbiota-targeted agents in secondary infection and non-secondary infection groups of H7N9 patients

**Characteristics**	**Secondary infection group**	**Non-secondary infection group**	**Healthy controls**
	**(*n*** **= 11)**	**(*n*** **= 14)**	**(*n*** **= 25)**
Median age (years)	64.5	48	58
Male:female (*n*)	9:2	8:6	17:8
Median hospital stay (days)	21.5	13	-^a^
Primary blood parameters at first follow-up:			
Mean WBC (SD, 10^9^cells per liter)	8.0 (3.5)	4.6 (3.3)	5.7 (1.3)
Mean CRP (SD, mg per liter)	61.7 (72.3)	42.8 (53.2)	-
ALT median (25^th^–75^th^ percentile, units per liter)	38 (25–50)	44 (19.5–114)	16 (12.5–21)
Antimicrobial usage:			-
Cephalosporins (*n*)	7	9	-
Carbapenems (*n*)	6	1	-
β-Lactamase inhibitors and cephalosporins (*n*)	4	3	-
β-Lactamase inhibitors and penicillins (*n*)	9	5	-
Quinolones (*n*)	10	8	-
Macrolides (*n*)	4	1	-
Vancomycin (*n*)	5	0	-
Tigecycline (*n*)	4	0	-
Oxazolidinones (*n*)	3	0	-
Phosphonomycin (*n*)	1	1	-
Antifungalagents (*n*)	1	0	-
Micro-ecological-targeted agents			-
CBM588 (*n*)	9	11	-
*Bacillussubtilis*- and *E. faecium*-coated enteric capsules (*n*)	1	1	-
Lactulose (*n*)	4	0	-
Infection with			-
*Canidia* (*n*)	7	0	-
*Klebsiella pneumoniae*, *n*	6	0	-
*Acinetobacter baumanii* (*n*)	5	0	-
*Pseudomonas* (*n*)	5	0	-
*Flavobacterium indologenes* (*n*)	1	0	-
*Staphylococcus* (*n*)	2	0	-
Infections with mixed culture (*n*)	7	0	-
Diarrhea (*n*)	3	0	-

*Abbreviations*: *WBC* white blood cell, *SD* standard deviation, *CRP* C-reactive Protein, *ALT* alanine aminotransferase, *CBM588*, *Clostridium butyricum* 588 strain MIYA-BM tablets.

^a^Not applicable.

### DNA extraction

DNA was extracted from the feces and bacterial precipitates using a Qiagen Stool Kit (Qiagen, Hilden, Germany) with a modified protocol for cell lysis [13]. DNA integrity was confirmed by agarose gel electrophoresis and ultraviolet photography with ethidium bromide staining.

### Qualitative and quantitative PCR to detect predominant intestinal bacterial population

Primers used were described in an earlier study [13] (Table 2). All oligonucleotide primers were synthesized by Takara (Dalian, China). Quantitative PCR was performed using a ViiA 7 Real-Time PCR System (Applied Biosystems, Foster City, CA, USA). Amplification reactions contained 10 μl of SYBR Green PCR Master Mix (Applied Biosystems, Warrington, UK), 300 nM each primer, and 300 ng of the respective crude template DNA or 1 μL of water (negative control) in a final volume of 20 μL. Each reaction was performed in duplicate, and a ΔC (t) < 0.5 between duplicates was required. Amplifications were performed with the following temperature profiles: one cycle at 95°C for 3 min, followed by 40 cycles of 95°C for 30 s, annealing for 40 s, and 72°C for 30 s. Fluorescence was measured at 80°C for 10 s after the extension phase of each cycle to avoid interference from primer dimers, secondary structure, or spurious priming. A final extension step of 72°C for 5 min was performed. The annealing and plate-reading temperatures for each primer pair are shown in Table 2. Following amplification, melting temperature analysis of PCR products was performed to determine the specificity of the PCR. The melting curves were obtained by slow heating at 0.1°C/s from 65°C to 95°C, with continuous fluorescence measurement.

**Table 2:** Primers used in this study

**Target group**	**Sequence (5′–3′)**	**Annealing temperature (°C)**
*Faecalibacterium prausnitzii*	GATGGCCTCGCGTCCGATTAG	58
	CCGAAGACCTTCTTCCTCC	
*Bifidobacterium* genus	GGGTGGTAATGCCGGATG	59
	TAAGCCATGGACTTTCACACC	
Lactic acid bacteria	AGCAGTAGGGAATCTTCCA	58
	ATTYCACCGCTACACATG	
*Enterococcus faecalis*	AACCTACCCATCAGAGGG	57
	GACGTTCAGTTACTAACG	
*Enterobacteriaceae*	CATTGACGTTACCCGCAGAAGAAGC	63
	CTCTACGAGACTCAAGCTTGC	
*Bacteroides-Prevotella* group	GAAGGTCCCCCACATTG	56
	CAATCGGAGTTCTTCGTG	

The copy number of rDNA operons of targeted bacteria in crude DNA templates was determined by comparison with serially-diluted plasmid DNA standards run on the same plate. The plasmid DNA standards were made from known concentrations of plasmid DNA that contained the respective amplicon for each set of primers, according to Bartosch et al. [14]. Determination of the detection limit of the assays was performed as described previously [15].

### PCR amplification of the 16S rDNA V3 region

The V3 variable region of 16S rDNA was amplified using a hot-start touchdown protocol with primers specific for conserved regions of the 16S rRNA gene [16]. The reaction mixture contained 800 ng of genomic DNA, 25 pmol of each primer, 0.2 μM dNTPs, 1 × Ex *Taq* buffer, and 2.5 U of Ex *Taq* polymerase (Takara). The final volume of the reaction mixture was adjusted to 50 μL with sterile deionized water. To minimize heteroduplex formation, five-cycle reconditioning PCR was conducted using 5 μL of amplification mixtures in fresh reaction mixture, as previously described [17]. Thermal cycling was performed in a TProfessional Thermocycler (Biometra, Göttingen, Germany). Concentrations were measured using a NanoDrop ND-1000 spectrophotometer (NanoDrop Technologies, Wilmington, DE, USA, USA).

### Denaturing gradient gel electrophoresis profiling

Parallel DGGE analysis was performed using the D-Code universal mutation detection system apparatus (Bio-Rad, Hercules, CA, USA) with 16 cm × 18 cm × 1.5 mm gels. Sequence-specific separation of the PCR fragments was obtained in 8% (wt/vol) polyacrylamide (acrylamide-N, N’-bisacrylamide, 37.5:1 (wt/vol)) gels in 1 × TAE buffer (40 mM Tris, 20 mM glacial acetic acid, 1 mM EDTA, pH 8.0). Denaturing gels contained a 35–75% gradient of urea and formamide, increasing in the direction of electrophoresis. A 100% denaturing solution contained 40% (vol/vol) formamide and 7 M urea. PCR fragments (20 μL) were loaded onto gels and electrophoresis was conducted at a constant voltage of 70 V and a temperature of 60°C for approximately 16 h. Following electrophoresis, gels were stained with SYBR green I (Sigma-Aldrich, Castle Hill, Australia) and photographed. Each DGGE gel included a standard reference (a randomly selected sample) in the middle and at both ends for digital gel normalization, and to allow comparison between gels.

DGGE profiles were digitally processed using BioNumerics software version 6.01 (Applied Maths, St-Martens-Latem, Belgium) following the manufacturer’s instructions. All profiles were compared using the band-matching tool, and uncertain bands were included in the position tolerance settings. Bands were allocated according to the parameters of Joossens et al. [18]. Principal components analysis (PCA) analysis and DGGE Bands Sequencing were performed according to a previous study [19].

## Results

### Retrospective analysis of microbiota-targeted treatment and secondary infection

**Figure 1:**
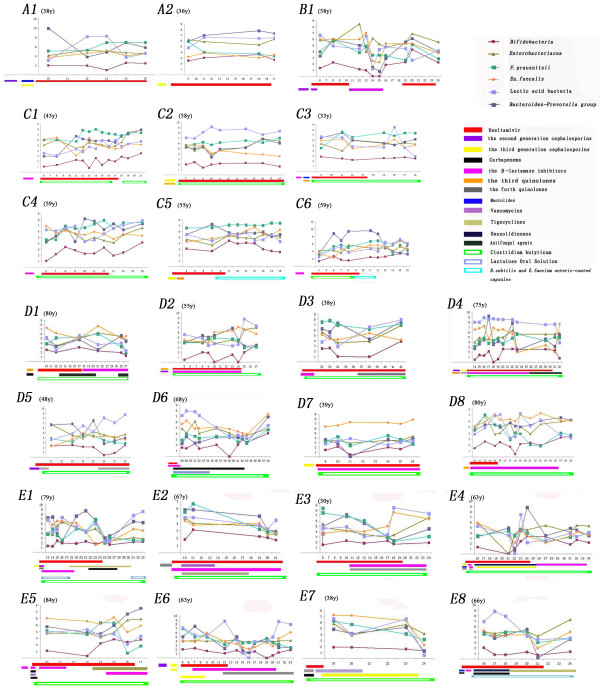
**Real-time monitoring of the changes in six predominant bacterial populations in 1 μg of fecal microbial DNA in 25 patients.** X axis, course of the treatment; Y axis, copies of 16 s rDNA operon from targeted bacteria in 1 μg of fecal microbial DNA. Patient age is given in brackets.

Early clinical experience with influenza virus H7N9 therapy showed that patients often developed acute exacerbation with bacterial superinfection. These serious complications could sometimes be fatal. Prophylactic selective decontamination of the digestive tract is suggested for increased survival of critically ill patients with H7N9 pneumonia. Therefore, all patients in our study received different broad-spectrum antibiotics in their district hospitals prior to transfer (Table 1 and Figure 1) to remove any pathogenic microorganisms. Most patients also received prophylactic selective decontamination of the digestive tract during the follow-up period. The antibiotics used in these patients were classified into nine groups according to molecular structure: cephalosporins (cefuroxime, cefoxitin, cefmetazole, ceftazidime, cefotaxime, ceftriaxone, cefdinir, ceftizoxime, and cefepime), carbapenems (imipenem and meropenem), β-lactam and β-lactamase inhibitors (cefoperazone sodium and sulbactam sodium, piperacillin-tazobactam, piperacillin sodium, and sulbactam sodium), quinolones (moxifloxacin, ciprofloxacin, and levofloxacin), macrolides (azithromycin and roxithromycin), vancomycin, tigecycline, oxazolidinones (linezolid), phosphonomycin, and antifungal agents (fluconazole and caspofungin). Probiotics, including *Clostrium butyricum*-, *Bacillus subtilis*-, and *Enterococcus faecium*-coated enteric capsules, and prebiotics, such as lactulose, were used in most patients in consideration of impaired intestinal colonization resistance and disturbance of microbiota caused by the broad-spectrum antimicrobial agents. Probiotic compounds were administered as two tablets, three times per day (containing ~10^7^ cfu/tablet for CBM588 and 10^8^ cfu for *B. subtilis* and *E. faecium* capsules). All microbiota-targeted agents were taken as advised, and administered by an expert team according to patient health status, the targeted disease, and the recipient’s perceived risks and willingness.

**Figure 2:**
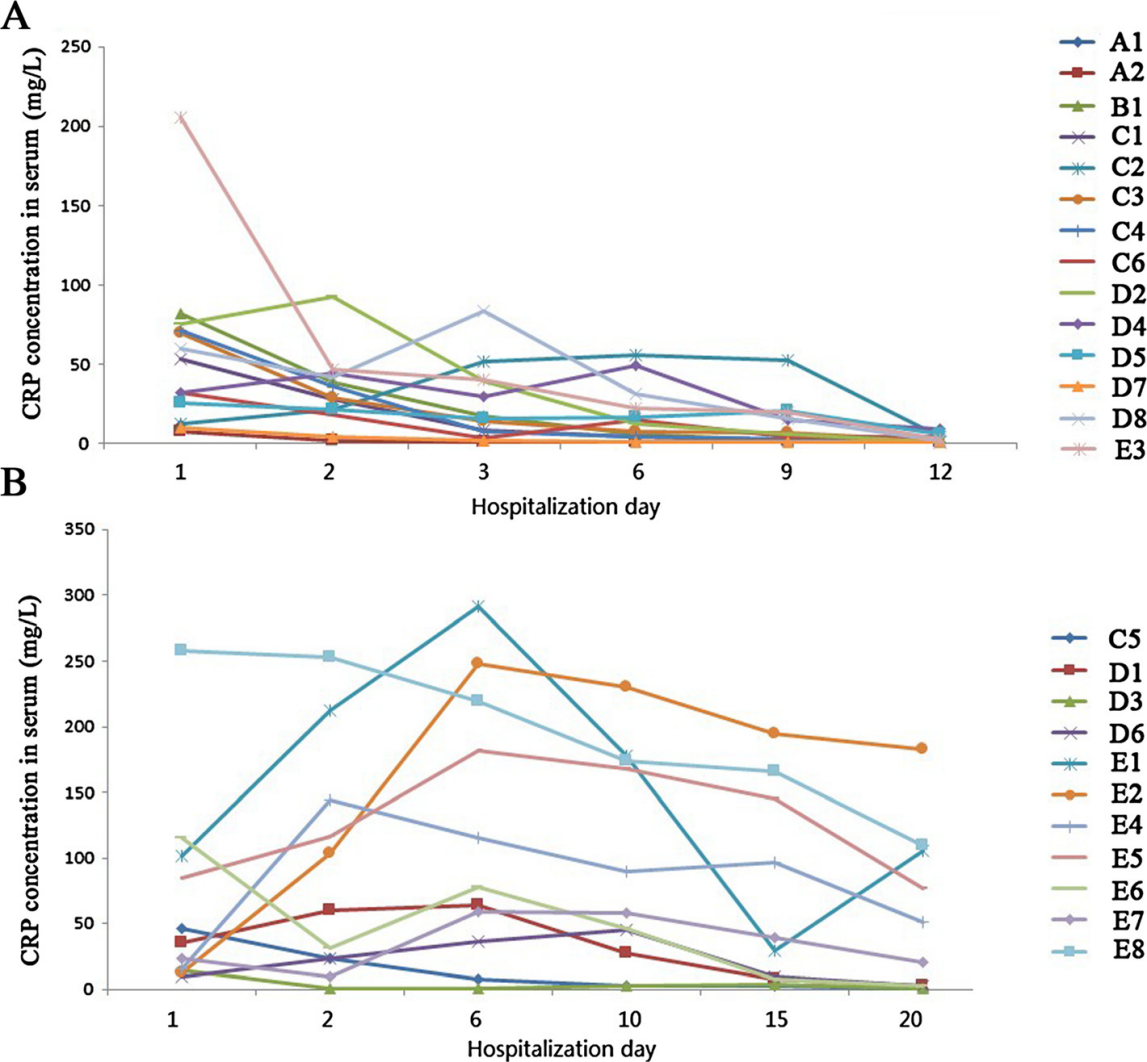
**The concentrations of C-reactive protein (CRP) in blood (mg/L). A**, Patients without secondary infection; **B**, patients with secondary infections.

Eleven patients (C5, D1, D3, D6, E1, E2, and E4–E8) acquired secondary bacterial infections during their stay in the intensive care unit for treatment of critical pneumonia. Eight of the eleven patients (72%) were older than 60 years of age. Pathogens were isolated from their clinical specimens. Six samples contained *Klebsiella pneumoniae* (isolated from sputum)*,* seven had *Candida* species (isolated from sputum), five had *Acinetobacter baumanii* (isolated from sputum), five had *Pseudomonas* species (isolated from sputum), two had *Staphylococcus* species (isolated from blood), and one had *Flavobacterium indologenes* (isolated from blood). Seven had mixed bacterial infections. Three patients from group E suffered from antibiotic-associated diarrhea caused by *Clostridium difficile*, which was cultured from stool samples.We also monitored the real time concentration of C-reactive protein (CRP), an early indicator of infection or inflammation, in the blood of each patient. Results showed that CRP tended to decrease in most H7N9 patients, particularly those without secondary infections (Figure 2), which suggested that the probiotics used were safe and did not cause an inflammatory state.

### Intestinal *Bifidobacterium*/*Enterobacteriaceae* ratio in patients with H7N9 infection

**Figure 3:**
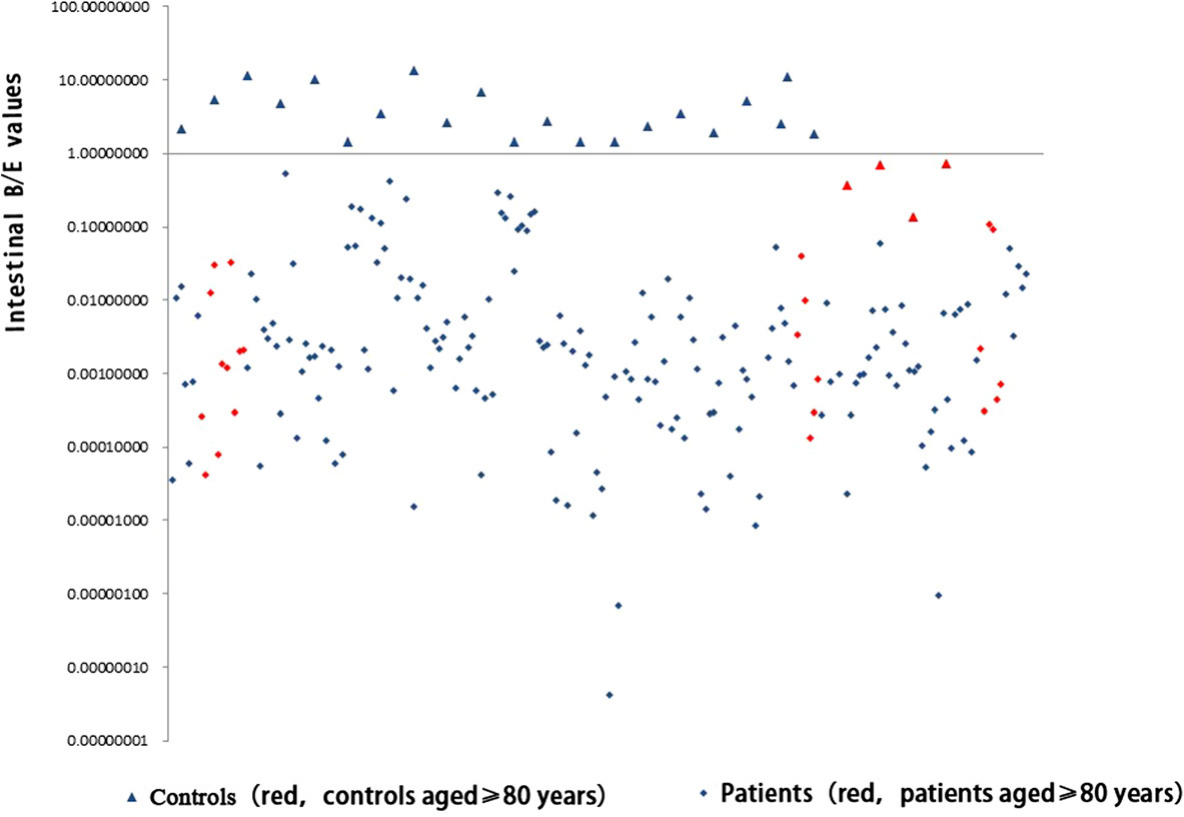
**Intestinal B/E ratio in patients with influenza virus H7N9 infection and healthy age- and sex-matched controls.** Ratios in most patients were < 1. Triangles, healthy controls; diamonds, patients. Red symbols denote ≥80 years of age.

All subjects were assessed for their intestinal *Bifidobacterium*/*Enterobacteriaceae* (B/E) ratio in the total fecal microbial DNA (Figure 3). B/E ratios of most healthy controls were ≥1, except for four individuals aged ≥80 years (red triangles in Figure 3) who had ratios < 1. In contrast, the ratios of all patients were < 1, and most were even smaller than in the elderly healthy controls.

### Real-time monitoring of the predominant intestinal microbiome

**Figure 4:**
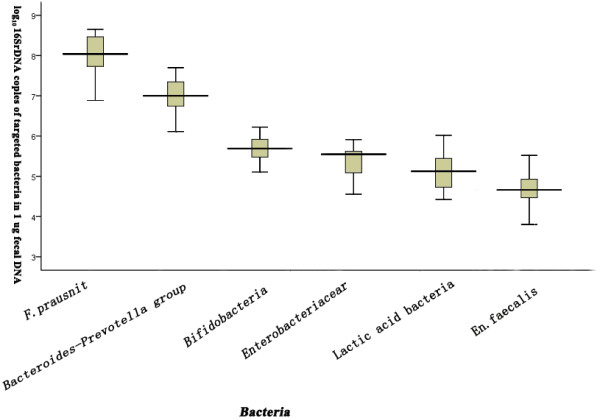
Levels of six predominant intestinal bacterial populations in healthy age- and sex-matched controls.

The quantification data of six predominant intestinal bacteria, *Bifidobacterium*, *Enterobacteriaceae*, *Bacteroides-Prevotella* group, *Faecalibacterium prausnitzii*, *Enterococcus faecalis*, and lactic acid bacteria, were expressed as log_10_ 16S rRNA gene copies in 1 μg fecal DNA. The levels of these bacteria in healthy controls are shown in Figure 4.

Two patients, A1 and A2, only received antiviral treatment at the follow-up stage, and antimicrobial treatment in the early stage had significantly decreased the *Bifidobacterium* population (A1 and A2 in Figure 1). The influence of antibiotics on fecal bacteria was clearly demonstrated in Patient B1, who had a marked decrease in the six bacterial groups during a 5-day period of antibiotic treatment in the follow-up period (B1 in Figure 1). The levels of these populations largely rebounded after antibiotic treatment ended. Notably, this patient had an increase in *Enterobacteriaceae* when taking piperacillin sodium and sulbactam sodium, which suggested an increase in resistant strains that are present in small numbers in the healthy intestinal tract. Patients C1–C6 received microbiota restoration therapies (C1–C6 in Figure 1) without any antibiotics after enrollment, and the population sizes of the six bacterial groups increased. However, the populations of *Bifidobacterium*, *F. prausnitzii*, and lactic acid bacteria showed slight variation in some cases. In particular, the six bacterial groups were relatively stable in Patient C5, who received *B. subtilis*- and *E. faecium*-coated enteric capsules; however, *Candida albicans* was isolated from the sputum during follow-up. *Bacillus subtilis* and *E. faecium* capsules replaced CBM588 tablets for 2 days in Patient C6, and the six bacterial populations did not show substantial fluctuations at the termination of probiotic treatment. Data from eight patients in Group D who received one type of antibiotic and CBM588 tablets showed that CBM588 decreased the impact of the antibiotics, and increased the bacterial populationduring the period of continual suppression of the same antibiotic (D1–D8 in Figure 1). Additionally, the *Bifidobacterium* population generally trended upward in most patients in Group D. For critically ill patients in Group E (E1–E8 in Figure 1), the populations of the six bacterial groups fell to their lowest levels and then began to rebound. Most patients in Group E (7/8) had secondary infection and diarrhea during the follow-up period.

**Figure 5:**
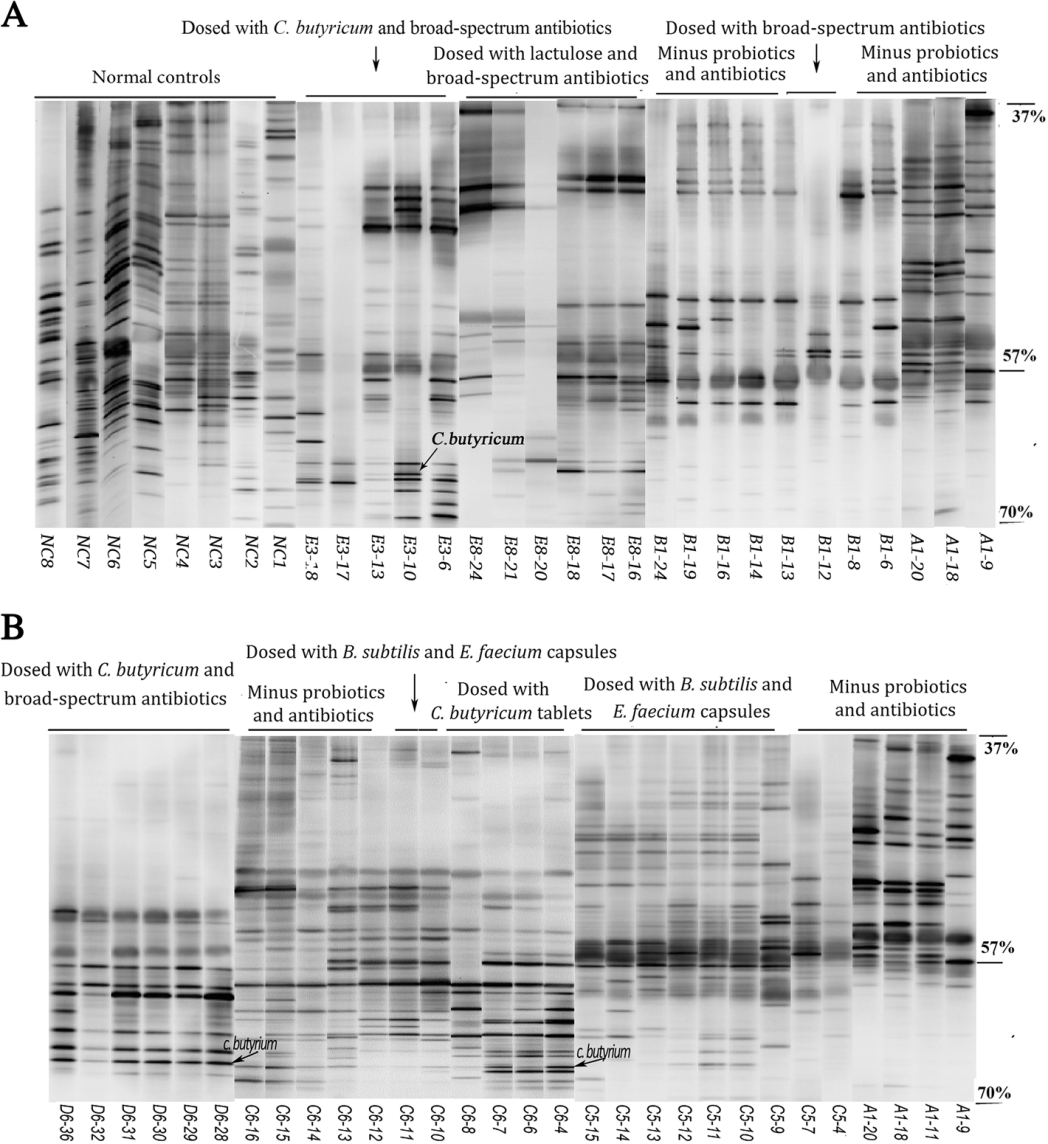
**DGGE profiles of representative predominant intestinal bacteria from each group (A1, B1, E3, E8, C5, C6, and D6) and seven healthy controls (NC1–NC7, age- and sex-matched with A1, B1, E3, E8, C5, C6, and D6, respectively).** The approximate denaturant concentrations in the gels were 37%, 57%, and 70%.

**Figure 6:**
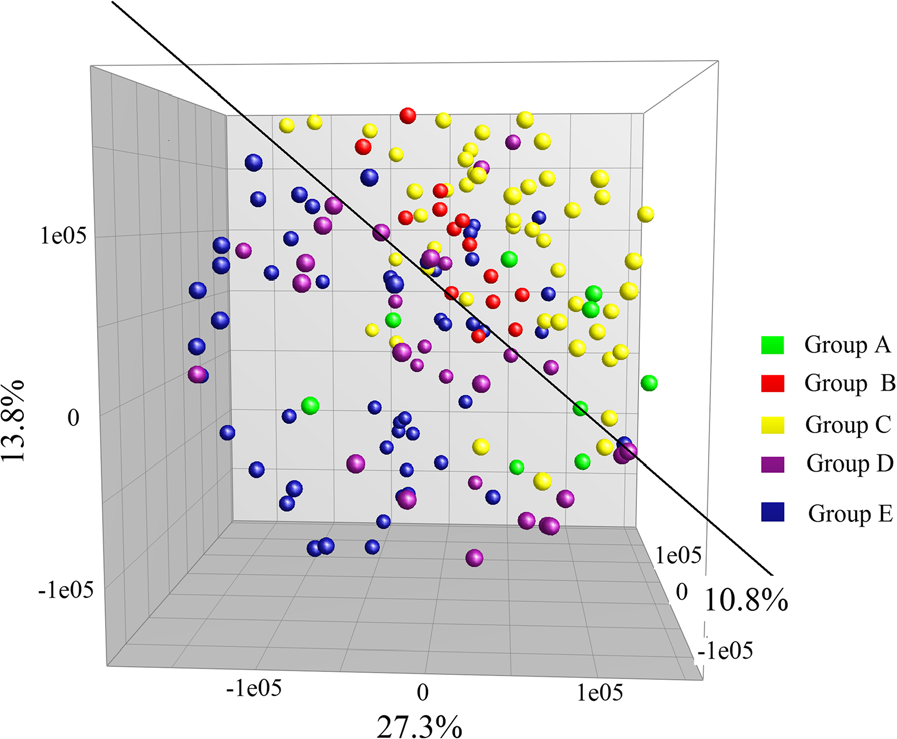
**Principal components analysisfor all DGGE profiles of patients.** Colored dots show the fecal microbiome profiles of five groups during follow-up. The plot is reoriented to maximize the variation among lanes along the first three principal components (contributions of 27.3, 13.8, and 10.8%, respectively) obtained from BioNumerics software.

Figure 5 shows the predominant intestinal microbiome profiles of representatives of each group. The predominant intestinal microbiome of control subjects was more evenly distributed, with higher species richness, than that of patients. The predominant bands in the profiles of B1 and A1 were distributed in the region where the denaturant concentrations had a range of 37–57%, but those dosed with *C. butyricum* for microbiota restoration therapy (E3, D6, and C6 in the early phase) were distributed in the 57–70% region, which revealed that *C. butyricum* might be involved in the recovery of bacteria with high GC% in the V3 region of 16S rDNA. Conversely, the profile of C5 indicated that *B. subtilis*- and *E. faecium*-coated enteric capsules might contribute to the restoration of the entire fecal microbiota. In particular, for C6, the most predominant bands were distributed in the 57–70% region in the early stages with *C. butyricum* treatment, but were distributed evenly throughout the entire gels when CBM588 was replaced by *B. subtilis* and *E. faecium* capsules in the middle stage. Furthermore, the profiles of most patients during the follow-up periods showed relative stability under the continual suppression by the same antibiotics. In contrast, the fecal DGGE profiles of E3 and E8 changed dramatically when new antibacterial agents were introduced during the microbiota restoration therapy.PCA analysis of DGGE profiles of all patients showed that the predominant intestinal microbiome of groups D and E differed from groups A, B, and C (Figure 6).

## Discussion

The gastrointestinal tract harbors more than 400 bacterial species, each having a specific niche and playing important roles in human health and disease [20]. Although it is difficult to define the normal intestinal microbiota, there is much evidence suggesting that the balance of beneficial and harmful bacteria (including opportunistic pathogens) is disturbed in many disease states [21-23]. Analysis of the fecal microbiota of healthy controls using DGGE in this study revealed that healthy gut microbiota was characterized by a relatively even distribution and high species richness and diversity. Most patients with influenza virus H7N9 infection had already received antibiotics in the early stages of the disease, and some of them had received repeated antibiotic therapy for secondary infections or prophylaxis against healthcare-associated infections during the microbiota restoration treatment period. In general, the more critically ill the patients were, the more antibiotics were used. Much evidence suggests that antibiotics may cause a profound imbalance within the intestinal microbiota [5] and lead to complications. Therefore, administering beneficial intestinal bacteria during selective decontamination of the intestinal microbiome was recommended by the therapy group in the present study. We monitored the populations of the six predominant intestinal bacterial groups, including three potential pathogens (*Enterobacteriaceae*, *Bacteroides-Prevotella* group, and *E. faecalis*), and three beneficial bacteria (*Bifidobacterium* species, *F. prausnitzii*, and lactic acid bacteria). The B/E values in this study revealed most young and elderly adult patients with H7N9 infection had imbalances between *Bifidobacterium* and *Enterobacteriaceae*. The disequilibrium could be caused by many factors, such as the aging process, influenza virus infection, antibiotics, and anorexia. Our results showed that *Bifidobacterium* was most sensitive to antibioticsuppressionand recovered poorly, even without antibiotic intervention. Additionally, the increasing antimicrobial drug resistance among *Enterobacteriaceae*[24] may largely account for the disequilibrium. *Bifidobacterium* inhibits the growth of various pathogens by several mechanisms, including production of antimicrobial agents and short-chain fatty acids, and competitive exclusion [25,26]. Thus, these species play an important role in preventing colonization of the intestine by pathogens. Furthermore, the *Bifidobacterium* population was often higher than or equal to that of the *Enterobacteriaceae* in most of the healthy controls in this and our previous study [23]. Our previous research showed that the B/E ratio is useful for monitoring microecological disruption in the intestine during the progression of liver disease [23].

Secondary infections in most critically ill patients were caused by exogenous rather than endogenous bacteria, most of which were respiratory infections caused by *Candida* species and multidrug-resistant *A. baumannii* and *K. pneumoniae*. Previous *in vitro* and *in vivo* studies have revealed that bacterial adherence to the surface of cells in the respiratory tract is enhanced by influenza virus infection, which contributes to the increased incidence of secondary bacterial infection in influenza patients [27]. As a result, microecologically targeted therapy was initiated to increase beneficial bacteria and reduce the population of potential pathogens to enhance resistance to intestinal colonization. Probiotics have been proposed to have the potential to modulate host innate immune responses outside the gastrointestinal tract, including the respiratory mucosa [28,29]. Furthermore, probiotic prophylaxis of ventilator-associated pneumonia wasreported to be successful [30]. The relative stability in the profiles of most patients under the continual suppression by the same antibiotics suggested that microbiota restoration therapy with probiotics might help to maintain the relative stability of the microbial community during the period of antibiotic treatment. We also observed that the effect of a combination of antibiotics and pre- or pro-biotic intervention on the predominant intestinal microbiome differed from that of antibiotic or probiotic intervention alone. Individual risk of infection is determined by the relationship between an individual’s epidemiological exposure and net state of immunosuppression. A weakened immune system in elderly patients might account for the severity of influenza infection and serious complications [31]. However, prophylactic use of probiotics was ineffective in elderly and critically ill patients in this study, and the elderly were stillat particularly high risk for developing secondary bacterial infection as a result of the primary influenza and the complex antibiotic therapies. The artificial liver technology largely contributed to the failure of pro- or pre-biotics to prevent respiratory infections in these patients. Artificial liver technology was used to remove inflammatory cytokines induced by endotoxins because of the use of broad-spectrum antibiotics, which was later confirmed to be a risk factor for critically ill H7N9 patients. In any case, our data showed that CBM588 failed to prevent respiratory infections in elderly and critically ill patients, and its effect on increasing levels of *Bifidobacterium* in the gut was unsatisfactory. There is a clear need for large volunteer studies on effective prophylaxis strategies for secondary respiratory infections in these elderly patients.

Results from this study demonstrated a transient stability of the intestinal microbiota under continual suppression with the same antibiotics in those patients treated with probiotics. Perhaps the most interesting results in our study were the different profiles of the intestinal microbiome that were restored by *C. butyricum*, *B. subtilis*, and *E. faecium* treatment. *Bacillus subtilis*- and *E. faecium*-coated capsules seemed to promote an even distribution in the fecal microbiota, and contribute to restoration of the entire fecal microbiota following antibiotic intervention. Microbiota restoration strategies with multiple probiotics may be more effective than single probiotics in restoring the entire intestinal microbiome. Future studies using properly designed clinical trials are required to support such claims and to uncover the mechanisms. Lactic acid bacteria also have protective effects against bacterial and viral infections in the gastrointestinal and respiratory systems [32], and likely contribute to the accelerated recovery of the innate immune response [28]. The qPCR results in this study showed that lactic acid bacteria recovered more quickly than the *Bifidobacteria* population, even under antibiotic suppression, which might be beneficial to H7N9 patients.

Another aim of probiotic treatment is to ameliorate inflammation in these patients. All patients with influenza virus H7N9 infection showed a sharp increase in inflammatory markers in the blood, caused by the primary and secondary infections. There are many reports of anti-inflammatory activity of probiotics, including CBM588 [10,33]. With regard to the assessment of microecological treatment strategies, treatment of H7N9 infection has previously focused on pathogenicity because of the translocation and virulence of probiotics [34], and conflict between antibiotics that kill bacteria and microecological regulatory agents that promote bacterial growth, so that the negative effects did not outweigh the benefits. CRP is normally present in trace levels in the serum, but increases rapidly in response to various infectious or inflammatory conditions [35]. In this study, we found no marked increase in CRP, and no cases of bacteremia and pneumonia, caused by probiotics in the patients.

Prophylaxis against secondary infection with antibiotics and probiotics is as important as antiviral treatment. It is necessary to integrate human and microbial genomic data sets to analyze the risk of human disease and achieve clinically effective strategies to maintain and promote human health [36]. Prevention and treatment of specific infections in these patients should be dictated by knowledge of the infecting microorganism and its sensitivity to antimicrobial agents, as well as the disruption of gut microflora balance during antibiotic treatment. Future studies on the composition of the dominant intestinal microbial populations in patients are important for successful infection prevention. Unfortunately, the number of patients in the present study was small, and all had received antibiotics prior to being transferred to our hospital. Well-designed animal trialsto investigate the use of CBM588 as an adjunct to enhance resistance to intestinal colonization are also needed.

## Conclusions

Our results demonstrated a potential clinical strategy for microbiota-targeted therapy in patients with influenza virus H7N9 infection. The mortality of critically ill patients in our hospital was lower than in other parts of China, which may be partly because of microbiota regulation therapy to compensate for impaired intestinal colonization resistance and to reduce bacteremia and sepsis. This study has noteworthy applications in the development of personalized therapeutic strategies by real-time monitoring of the predominant intestinal bacteria using qPCR and DGGE, which is fast, cheap, and comparatively less laborious than high-throughput sequencing technologies. Additionally, the present study is the first to use intestinal B/E ratio for rapid nonintrusive assessment of intestinal colonization resistance and microbiota-regulated therapy in patients with influenza virus H7N9 infection.

## Abbreviations

DGGE: Denaturing gradient gel electrophoresis; CRP: C-reactive protein; qPCR: Quantitative polymerase chain reaction; CBM588: Clostridium butyricum 588 strain MIYA-BM tablets.

## Competing interests

The authors declare that they have no competing interests.

## Authors’ contributions

HL and CZ helped design the study, collected and analyzed data, and drafted the manuscript. GQ and XH performed the experiments and acquired data. ZH prepared the figures. CC, WL, HG and YY had roles in recruitment, field investigation, and were involved in data analysis, interpretation and write-up. LL designed the study and critically revised the manuscript. All authors read and approved the final manuscript.

## Pre-publication history

The pre-publication history for this paper can be accessed here:

http://www.biomedcentral.com/1471-2334/14/359/prepub
